# Survey of Indian Dental Professionals Regarding the Use of Computer-Aided Design/Computer-Aided Manufacture (CAD/CAM) Technology

**DOI:** 10.7759/cureus.40392

**Published:** 2023-06-13

**Authors:** Vivek Choukse, Anuja Kunturkar, Ashwin Nirmal Aidasani, Abhilasha Masih Gottlieb, Ruchi Agrawal, Pratik P Bumb

**Affiliations:** 1 Department of Prosthodontics and Crown and Bridge, Dr. Hedgewar Smruti Rugna Seva Mandal's Dental College and Hospital, Hingoli, IND; 2 Department of Prosthodontics and Crown and Bridge, Teerthanker Mahaveer Dental College and Research Centre, Moradabad, IND

**Keywords:** indian dentist, digital dentistry, survey, dentist, cad cam

## Abstract

Objective

In recent years, digital processes like computer-aided design/computer-aided manufacture (CAD/CAM) have been implemented in dentistry. On the use and reporting of this technology by dentists, there is no published information. The goal of this study was to determine whether CAD/CAM technology had infiltrated Indian dentistry practices and to look into the relationship between various demographic parameters and respondents' answers about using or not using this technology.

Materials and methods

A sample of Indian dentists, both users and non-users of CAD/CAM, were sent about 500 online surveys. It sought to shed light on the usage style, materials, advantages, and limitations of CAD/CAM dentistry, as well as their perceived advantages and access hurdles. Statistical analysis was conducted to determine the impact of numerous demographic factors, including country of employment, dentist experience, educational attainment, and the nature of the job.

Results

There were 132 total replies that were finished. The majority of respondents didn't use any aspect of a digital workflow, and the biggest obstacles to using CAD/CAM were the upfront expenses and a lack of perceived advantages over traditional techniques. The likelihood of using CAD/CAM technology was highest among dentists who primarily performed private practice (P<0.0001). A third of users thought that their training was insufficient, even though the majority of users were either self-taught or schooled by firms. The majority of respondents (60.6%) believed that CAD/CAM would have a significant future role.

Conclusion

Most respondents said they had never used any aspect of a digital process. Although most dentists who responded to the study thought CAD/CAM would play a significant role in the future, the majority were still interested in integrating it into their workflow. Dentists continue to have certain concerns regarding the chairside CAD/CAM restorations' quality.

## Introduction

Different prostheses, such as crowns, fixed dental prostheses (FDPs), and removable dental prostheses, are fabricated from a variety of dental materials using a variety of dental laboratory processes. An increase in the demand for esthetics and biocompatibility has led to the development of various metal-free materials [1]. Dental ceramics have been in use in dentistry for the last 100 years [2]. The use of all ceramic prostheses has rapidly increased in the past few years. In past years, traditional laboratory methods were used for fabrication [3]. The conventional method of fabrication is time-consuming and technique-sensitive. This led to the development of high-strength ceramics that can be used only in conjunction with computer-aided design/computer-aided manufacture (CAD/CAM) [4-6]. CAD/CAM restorations have numerous advantages over traditional methods, including ease of fabrication, speed, and quality of restoration [1]. CAD/CAM has become an increasingly popular part of dentistry over the past 25 years. An intraoral scanner is used to build 3D representations of your teeth and gums over the tooth preparation. We can notice more details faster owing to the exceptional accuracy of these digital dental imprints. Contrarily, the intraoral scanner is a thin gadget that doesn't make patients uncomfortable or uneasy. It is non-invasive, quick, and accurate. We will transfer the digital dental imprints we take to our computer, and then your crown or bridge will be made using milling in a single appointment. It is rapidly used in the fabrication of inlays, onlays, veneers, crowns, fixed partial dentures, implant abutments, and even full-mouth reconstruction [7].

CAD/CAM dentistry has grown in Delhi-NCR, a healthcare hub. CAD/CAM-equipped dental clinics and laboratories have improved dental treatments, prosthetic restorations like crowns, bridges, and veneers, and patient turnaround time. This has improved dental patient satisfaction and revenue. Mumbai, India's largest city, has a growing dental industry with many clinics and laboratories. CAD/CAM technology lets dentists do same-day restorations, saving time and appointments [4]. This convenience and efficiency have increased patients and boosted regional dental practices' profits. Bengaluru, known for its technology and healthcare, has adopted CAD/CAM dentistry. City dental clinics and laboratories use CAD/CAM systems to create precise, aesthetic dental restorations. This increased patient flow, dental tourism, and the local economy. CAD/CAM technology has improved dental treatment options in Chennai's strong dental sector. CAD/CAM systems allow dentists to create custom crowns and bridges with precise fit and natural aesthetics. This has drawn patients from across the region, boosting Chennai's dental industry. Dental tourism has grown in Kochi, Kerala. CAD/CAM technology helps international dental patients receive high-quality care. Advanced CAD/CAM systems in dental clinics and laboratories enable efficient and precise restorations, attracting patients and boosting the local economy. CAD/CAM benefits Hyderabad's dental industry. For implant restorations, dental prosthetics, and orthodontic appliances, local dentists use digital workflows and CAD/CAM systems. CAD/CAM technology has improved treatment outcomes, reduced chair time, and expanded service offerings, helping the dental sector grow [5-7].

Despite improvements and benefits, there aren't many research on dentists' awareness and usage of CAD/CAM. The purpose of this survey was to determine whether CAD/CAM technology had infiltrated Indian dentistry practices' and to look into the relationship between key demographic variables and the responses about use or nonuse of this technology. 

## Materials and methods

To determine the use and penetration of CAD/CAM technology in Indian dentistry practices and to explore the relationship between different demographic parameters and the responses about the use or non-use of the technology, a brief online survey of 20 questions was devised and conducted in India. The study was conducted at Dr. Hedgewar Smruti Rugna Seva Mandal's Dental College and Hospital, Hingoli, India. An online approach was used to maximize responses, make collection and analysis easier, and decrease costs. The sample size was calculated using the G Power Version 3.1.9.6 program (Franz Faul University, Keil). Based on the 95% confidence interval, 8% type I error, and a response distribution of 50%, the sample size was estimated to be 126, and taking into account the 5% attrition rate, the sample size was increased to 132.

A self-designed, closed-ended questionnaire was administered to the study sample. A questionnaire consisting of 20 questions was divided into three parts. The Cronbach's alpha value was 0.8, and the content validity index had a value of 1 for the questionnaire that was used. (Table 1)

**Table 1:** Questionnaire used in the study CAD: computer-aided design; CAM: computer-aided manufacture; CEREC: chair-side economical restoration of esthetic ceramic

How many years have you practiced as a dentist?
0-10
10-20
More than 20
Area of practice?
Urban
Rural
How much formal training have you had?
General dental practitioner
Specialist prosthodontist
Post graduate student
Other:
Is the work that you do: Government/Private
Predominantly government
Predominantly private
Mix
Do you use any aspect of CAD/CAM in your workflow?
Yes
No
Have used it in the past but no longer use it currently?
For CAD CAM Users
How long have you been using CAD/CAM for?
0-5 Years
6-10 Years
11-15 Years
>15 Years
What precipitated your move towards a CAD/CAM workflow?
To reduce lab fees
To improve productivity
To improve quality
To use new dental materials which can only be fabricated with CAD/CAM, eg zirconia
To keep up with technology
To improve communication with the laboratory
As a marketing tool for patients
Other:
Which of these aspirations do you think you have achieved with CAD/CAM?
Reduction in bills
Improvement in quality
Improvement in productivity
It has been a good marketing tool for patients
Kept up with technology in dentistry
Improvement in communication with the laboratory
Other:
Which aspects of the digital workflow do you use?
Chairside CAD/CAM, for example, CEREC
Laboratory scanning of impressions or casts
Computer-aided- design (CAD by laboratory or specialist milling center)
Computer-aided manufacturing (CAM by laboratory or specialist milling center)
Other:
Where did you undertake your CAD/CAD system training?
Companies providing CAD/CAM system
Private courses
Self-taught or taught by other users, etc.
Other:
Did you feel your CAD/CAM training was sufficient?
Yes
No
Do you feel that the availability of CAD/CAM has affected your clinical decision-making?
Yes
No
Has CAD/CAM led to changes in your use of dental materials?
Yes
No
What materials do you regularly use with CAD/CAM?
Strengthened ceramics, eg E.max
Polycrystalline ceramics, for example, zirconia/alumina-based
Composite
Metals
Other:
What are the least satisfactory aspects of your CAD/CAM finished restorations?
Marginal fit
Contact points
Occlusion
Aesthetics
I do not see that these restorations have a weakness
Other:
For non-CAD/CAM users
Why do you not use CAD/CAM?
High costs
Inferior quality of restorations
I am not very technologically aware
Do not see that there are any advantages over conventional techniques
Other:
Why did you stop using CAD/CAM (past users)?
Higher costs
Inferiority quality of restorations
Could not learn how to use the system
Did not see that there are any advantages over conventional techniques
Other:
Would you be interested in incorporating CAD/CAM as part of your workflow?
Yes
No
Do you think that CAD/CAM has a big role in the future of dentistry?
Yes
No

One part aimed to gather data regarding the demographic characteristics of the participating dentists, i.e., age group, sex, area of expertise, years since graduation, and their use of CAD/CAM in practice. The second part was focused on the CAD/CAM users and their knowledge and applications. The third part focused on non-CAD/CAM users. Most of the questions were multiple-choice questions, but the option was still given to give a free-response answer.

The data analysis was done by IBM SPSS Statistics for Windows, Version 25.0 (Released 2017; IBM Corp., Armonk, New York, United States). Potential relationships were investigated using statistical analysis and chi-squared testing. A significance level of 0.01% was used to reduce the effects of multiple tests. Therefore, throughout the studies, any p-values < 0.01 are taken to be significant.

## Results

**Figure 1:**
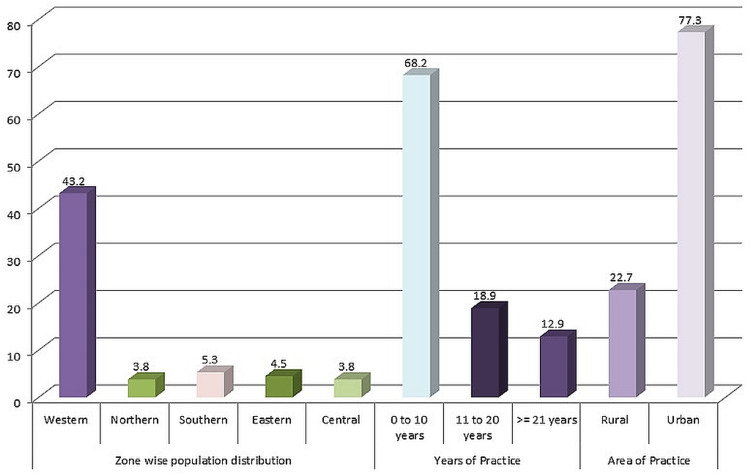
Demographic characteristics of the population

During a six-month period, a total of 132 dentists participated in the survey. Out of these 57 were from western areas, five from northern, seven from southern, six from eastern, and five from central. The majority of the participants were from Western areas (43.2%). Most of them (68.2%) had a working experience of zero to ten years. More than half of the participants were practicing in urban areas (77.3%). (Figure 1)

**Figure 2:**
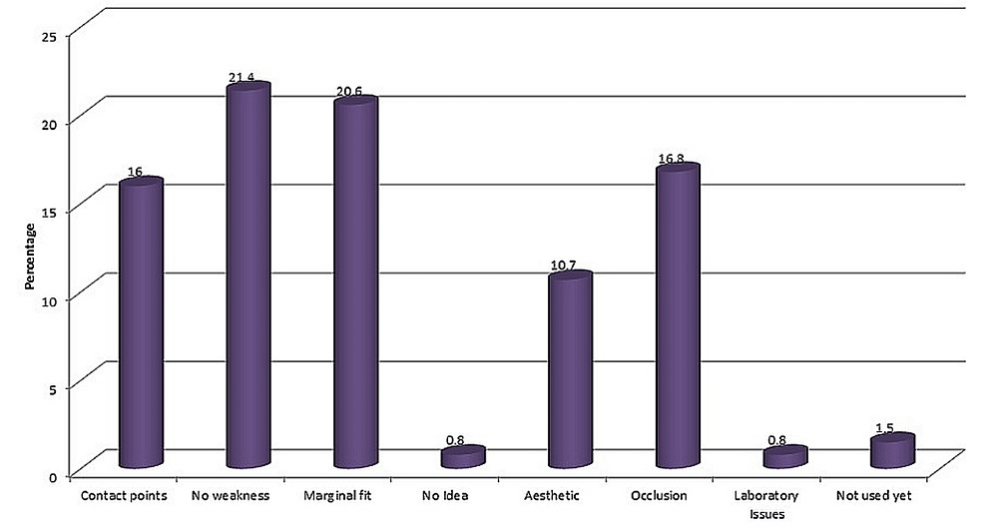
Least satisfactory aspects

About 50% of postgraduates had received formal training. 30.5% of the respondents have learned the usage either by themselves or from another user. 50.8% of participants have never used CAD/CAM in practice. When the participants are asked about the least satisfactory aspects of using CAD/CAM, the majority of the responses include marginal fit and contact points. (Figure 2)

**Figure 3:**
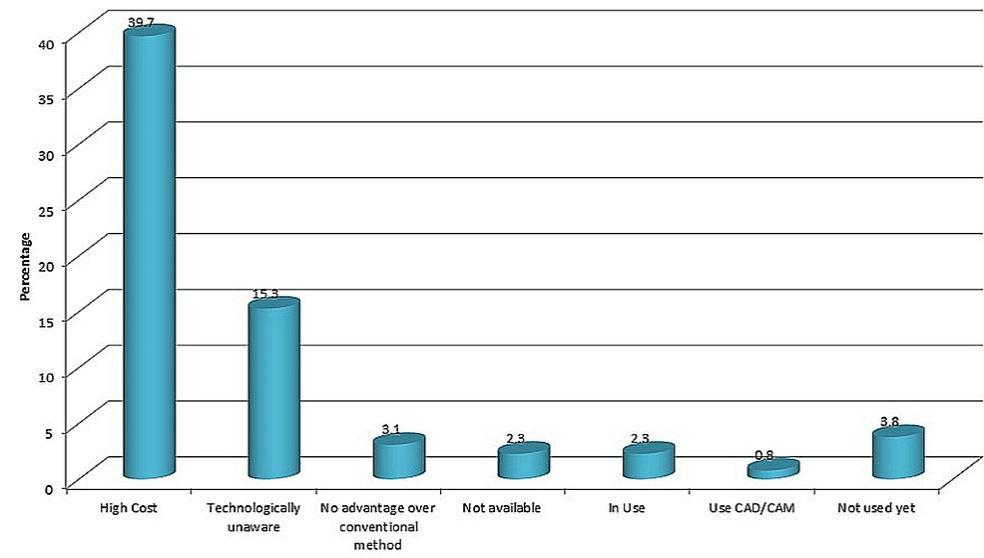
Reasons for not using CAD/CAM CAD: computer-aided design; CAM: computer-aided manufacture

The main reason for not using CAD/CAM was the high cost. The second reason for not using CAD/CAM was a lack of awareness of the technology and its advantages. Among the past users of CAD/CAM, the main reason for stopping the use of CAD/CAM in their practice was the higher costs. (Figure 3)

**Figure 4:**
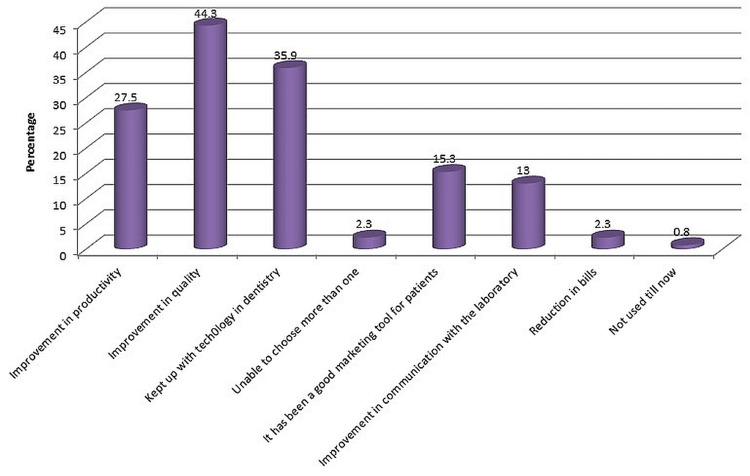
Aspirations achieved using CAD/CAM CAD: computer-aided design; CAM: computer-aided manufacture

Over 40.9% of the respondents who used CAD/CAM in any capacity in their workflow did so within the last five years. A higher likelihood of using CAD/CAM was associated with more postgraduate training (P = 0.0001). The majority of respondents (60%) thought that CAD/CAM would play a significant role in the future and were therefore interested in integrating CAD/CAM into their workflow. According to half of the respondents, it has also changed how dental materials are used and altered clinical decision-making. A total of 40.9% have used CAD/CAM for zero to five years in their practice. Only 16.7% of participants thought their training was satisfactory. (P=0.0001). (Figure 4)

Half of the CAD/CAM users stated that they had achieved improvements in the quality of the restorations through the use of CAD/CAM. There were only 0.8% of the respondents who had never used CAD/CAM. And the majority of the respondents have used CAD/CAM at a laboratory or specialized milling center.

## Discussion

Research is primarily based on data collection. The most common method of data collection is through surveys [8]. Data collection methods can be categorized into two categories: manual and electronic. The manual method of data collection is basically paper-based. There are numerous challenges to the manual method of data collection. The difficult part is converting the data to an electronic format for processing and analysis. The procedure is inefficient, expensive, and time-consuming and often results in inadequate data [9]. With the advances in science and technology, it is easier to do electronic data collection. There are primarily three different methods of electronic data collection: computer-administered surveys, electronic mail surveys, and web surveys [10].

In this study, a web-based survey was used. Google Forms are the ideal method for sending out a short questionnaire, charting the results, or exporting them for analysis to a spreadsheet [8]. This allows for a larger sample size, decreased costs for the project, and a relatively shorter period of time. However, the response rate could have been lower because of a number of factors, including an unused email address, a lack of interest in participating in the survey, and the inapplicability of the survey to other dentists [1]. The response of the dentists can be improved by a number of methods, like providing prizes and lucky draws, reminders, and for a longer duration of time. Although the response rate was not higher, the level of significance was adjusted to allow for significant results.

The majority of the participants were from western areas of India. The vast majority of the respondents (about 70%) had a working experience of zero to ten years, mainly in urban areas. The majority of them were in private practice. Thus, this data suggests that the majority of the participants were young dentists. The majority of the participants (less than half) have only recently begun using CAD/CAM in their practices. This data demonstrates that for the majority of dentists, CAD/CAM is still a relatively new phenomenon in the dentistry industry. In India, a statistical analysis of CAD/CAM has never been done before. Meaningful comparisons of this study with the existing literature cannot be made due to the lack of comparable investigations. The majority of respondents (roughly half) had training after graduating, and they were more likely to use CAD/CAM in their work. Almost equally as many respondents utilized CAD/CAM in their work.

The majority of dentists have agreed that the CAD/CAM technology has affected their decision-making and has resulted in an improvement in the quality of the restoration. This result is in accordance with the available literature, which shows that CAD/CAM restorations can be fabricated with good quality, are cost-effective, and are time-effective [11,12]. In terms of dental materials, a greater percentage of the respondents used polycrystalline ceramics. Whereas in more complicated procedures, specialists are more likely to use precious alloys, and these precious alloys cannot be used for CAD/CAM fabrication. The most conservative preparation is required for gold crowns, and thus they still remain the gold standard in terms of performance and durability [13,14]. About 30% of the respondents were self-taught or taught by other users. This is a clear indication of a lack of adequate training in dental education, so formal training must be provided as a part of the curriculum. The majority of the dentists highlighted that there is no weakness in the technology. 

In their professional lives, half of the participants have never used CAD/CAM. The greater expenses were the main deterrent to not employing CAD/CAM in their practice. Lack of expertise and awareness of the technology's benefits was another typical excuse for not using CAD/CAM. The present body of research, however, demonstrates that restorations created using a computerized methodology are just as functional and durable [15,16]. More than half of non-users expressed interest in implementing CAD/CAM in the future and concurred that it will play a significant part in their practices.

The limitations of the study were limited replies and questions. In the current study, more parameters may have been included in the questionnaire to produce higher-quality outcomes. Additional developments in implant technology, aesthetic dentistry, and maxillofacial prosthetics may have been incorporated to assess the level of knowledge and awareness among Indian dentists.

## Conclusions

The use of computer-aided design and manufacturing (CAD/CAM) technologies in dentistry has rapidly increased. Dentistry's uses for this revolutionary technology are growing and positive. The majority of respondents did not use CAD/CAM in their practice, and the main reasons for this were identified as greater initial expenses and a lack of knowledge. The majority of them concur that CAD/CAM will play a significant role in the future.
